# Mutational profiles of spontaneous and radiation-related mammary carcinomas in a rat model of Brca1 haploinsufficiency

**DOI:** 10.1038/s41598-026-41240-9

**Published:** 2026-02-24

**Authors:** Yuzuki Nakamura, Kazuhiro Daino, Atsuko Ishikawa, Shizuko Kakinuma, Yukiko Nishimura-Yano, Kento Nagata, Masaru Takabatake, Mayumi Nishimura, Tomoji Mashimo, Kazumasa Inoue, Tatsuhiko Imaoka

**Affiliations:** 1 Department of Radiation Effects Research, Institute for Radiological Science, National Institutes for Quantum Science and Technology, Chiba, Japan; 2 Department of Radiological Sciences, Graduate School of Human Health Sciences, Tokyo Metropolitan University, Tokyo, Japan; 3 Japan Society for the Promotion of Science Research Fellow, DC, Tokyo, Japan; 4 Institute for Quantum Life Science, National Institutes for Quantum Science and Technology, Chiba, Japan; 5 Department of Radiobiology, Institute for Environmental Sciences, Aomori, Japan; 6 Laboratory Animal Research Center, Institute of Medical Science, The University of Tokyo, Tokyo, Japan

**Keywords:** BRCA1, Breast cancer, Haploinsufficiency, Radiation, Whole-exome sequencing, Cancer, Genetics, Molecular biology, Oncology

## Abstract

Female carriers of a heterozygous germline mutation in *BRCA1*/*2* have a high risk of breast cancer. Although recent research has suggested that genomic instability via BRCA1/2 haploinsufficiency contributes to the early phase of BRCA-associated carcinogenesis, insights into the role of BRCA haploinsufficiency in carcinogenesis are lacking. We previously reported that the *Brca1*^L63X/+^ rat, a model of Brca1 haploinsufficiency carcinogenesis, exhibits a significantly higher incidence of mammary carcinomas than wild-type rats exposed to ionizing radiation; notably, the carcinomas retained a wild-type *Brca1* allele. To explore the mutation spectrum underlying Brca1 haploinsufficiency, we performed whole-exome sequencing of spontaneous and radiation-associated mammary carcinomas in wild-type and *Brca1*^L63X/+^ rats. Mammary tumors from wild-type and *Brca1*^L63X/+^ rats did not differ significantly regarding the number of somatic single-nucleotide variants (SNVs), small insertions/deletions (InDels), or frequency of copy-number variants (CNVs). The radiation-associated carcinomas of *Brca1*^L63X/+^ rats had significantly fewer identifiable cancer-driver mutations induced by SNVs and InDels than those of wild-type rats; moreover, irradiated *Brca1*^L63X/+^ rats tended to have more carcinomas with no detectable cancer-driver mutations via SNVs, InDels or CNVs. Thus, Brca1 haploinsufficiency contributes to breast carcinogenesis by bypassing the generation of cancer-driver mutations that would otherwise occur via accumulation of nonsynonymous mutations and CNVs.

**Supplementary Information:**

The online version contains supplementary material available at 10.1038/s41598-026-41240-9.

## Introduction

Breast cancer is the most common cancer in women^1^. Germline pathogenic variants have been estimated to cause 5−10% of female breast cancers, with *BRCA1/2* variants being the most common^2–5^. By age 80 years, female carriers of a heterozygous germline mutation in *BRCA1/2* have an approximately 70% higher cumulative risk of developing breast cancer than do noncarriers^6,7^, and they also have increased estimated risks for cancers of the ovary, pancreas, and stomach^6^. BRCA1/2 are tumor suppressors involved in repair of DNA double-strand breaks via homologous recombination^8^. Previous studies have described that exposure to diagnostic or therapeutic ionizing radiation at a young age increases breast cancer risk in female carriers of a *BRCA1/2* pathogenic variant^9–12^, suggesting a high susceptibility to carcinogens that can induce DNA double-strand breaks.

The mechanisms underlying BRCA-associated carcinogenesis are not fully understood. For a long time, a complete lack of BRCA1/2 function due to loss of the wild-type (WT) *BRCA1*/*2* allele via loss of heterozygosity (LOH) has been believed to cause carcinogenesis owing to the fact that *BRCA1/2* LOH was frequently observed in breast cancers with *BRCA1*/*2* germline mutations^2^. However, recent studies suggest that tumorigenesis can occur without LOH of *BRCA1*/*2*, indicating that BRCA1/2 haploinsufficiency contributes to BRCA-associated carcinogenesis^13^. For carriers of a *BRCA1*/*2* pathogenic variant, LOH of *BRCA1*/*2* was not observed in at least 10% of breast cancers^14^, and pancreatic cancer showed no frequent LOH of *BRCA2*^15^. Furthermore, even breast cancers with *BRCA1*/*2* LOH in carriers exhibited substantial heterogeneity of *BRCA1*/*2* LOH; not all cancer cells in a tumor had lost the WT *BRCA1*/*2*^14,16,17^. Impaired DNA repair capacity and genomic instability induced by BRCA1 haploinsufficiency were observed in studies using normal cells^18,19^. These findings indicate that LOH of *BRCA1*/*2* is a late event of BRCA-associated carcinogenesis and that Brca1 haploinsufficiency is involved in its early phase. Thus, elucidation of the role of BRCA1/2 haploinsufficiency in tumorigenesis will be a key advance for developing strategies for chemoprevention and treatment for carriers of a *BRCA1*/*2* germline variant. However, knowledge is lacking in this regard because no animal models are available that recapitulate *Brca1*/*2* haploinsufficiency^13^; notably, murine *Brca1* heterozygous mutant models do not show an increased risk of developing carcinomas^20–22^. We previously established a *Brca1*^L63X/+^ rat with a heterozygous germline *Brca1* variant, which exhibited a significantly higher incidence of mammary carcinomas than WT rats in which DNA damage had been induced by ionizing radiation (with no increased incidence in nonirradiated *Brca1*^L63X/+^ compared with nonirradiated WT rats); of note, the mammary carcinomas showed no evidence of inactivation of the WT *Brca1* allele^23^. Therefore, the *Brca1*^L63X/+^ rat is the first model to show an increased risk of mammary carcinoma under Brca1 haploinsufficiency^23^. Thus, investigations using *Brca1*^L63X/+^ rats would be expected to provide new insights into the role of BRCA1 haploinsufficiency in BRCA-associated carcinogenesis.

Findings of BRCA1/2 haploinsufficiency from the aspect of the mutation spectrum of breast cancer are lacking. Although one study analyzed the mutation spectrum of breast cancers with *BRCA* mono-allelic loss in carriers of a germline mutation, the evidence was insufficient owing to a small sample size^2^. Otherwise, no reports exist concerning the mutation profiles of radiation-induced breast cancers in carriers of a *BRCA1*/*2* pathogenic variant. To address this deficiency, we performed whole-exome sequencing to characterize the mutation spectrum of spontaneous and radiation-associated mammary carcinomas arising in *Brca1*^L63X/+^ rats.

## Results

### Characteristics of mammary carcinomas used for whole-exome sequencing

Whole-exome sequencing was performed on 38 mammary carcinomas including spontaneous (*n* = 9) and radiation-associated (*n* = 9) carcinomas that developed in WT rats and spontaneous (*n* = 9) and radiation-associated (*n* = 11) carcinomas that developed in *Brca1*^L63X/+^ rats (see Supplementary Table S1 online). These carcinomas were derived from a previous animal experiment that reported *Brca1*^L63X/+^ rats had a significantly higher risk of developing mammary carcinoma than WT rats after exposure to 2 Gy radiation at age 3 weeks (*P* = 0.04), with no increased incidence of spontaneous carcinomas in *Brca1*^L63X/+^ rats compared with WT rats (Supplementary Fig. S1)^23^. Due to the small number of radiation-associated carcinomas from WT rats, additional carcinomas obtained from another animal experiment^24^ were used (see Materials and Methods for details). There were no significant differences between carcinomas of WT and *Brca1*^L63X/+^ rats in terms of age at detection, age at euthanasia and autopsy, tumor weight, positive-cell rates for ERα, PgR and Ki-67, or Erbb2 score (Table 1, Supplementary Table S1).

**Table 1:** Characteristics of mammary carcinomas for whole-exome sequencing.

Group	*n* ^a^	Age at detection (weeks)	Age at autopsy (weeks)	Tumor weight (mg)	ERα^+^ cells (%)	PgR^+^ cells (%)	Erbb2 (score)	Ki-67^+^ cells (%)
Non-irradiated WT	9	57.7 ± 17.7	73.8 ± 20.4	5.9 ± 7.4	30.4 ± 23.7	31.3 ± 19.8	0.7 ± 0.9	20.1 ± 10.7
Non-irradiated *Brca1* ^L63X/+^	9	74.8 ± 27.9	88.0 ± 24.9	9.8 ± 16.4	14.3 ± 13.4	27.7 ± 26.0	1.8 ± 1.0	22.2 ± 14.4
Irradiated WT	9	51.0 ± 24.0	73.3 ± 19.8	28.7 ± 37.8	7.4 ± 6.1*	19.1 ± 21.8	2.3 ± 0.5*	17.0 ± 13.0
Irradiated *Brca1* ^L63X/+^	11	69.5 ± 16.7	76.6 ± 17.0	4.2 ± 3.5	9.4 ± 6.9*	32.1 ± 17.7	2.1 ± 0.5*	27.6 ± 12.9

^a^*n*, number of mammary carcinomas (i.e., number of rats). *P < 0.05 versus corresponding untreated WT group. The Steel-Dwass test was used to compare ages and Erbb2 scores among groups. The Tukey-Kramer test was used to compare weights and percentages of positive cells. All data represent the mean ± SD.

**Fig. 1:**
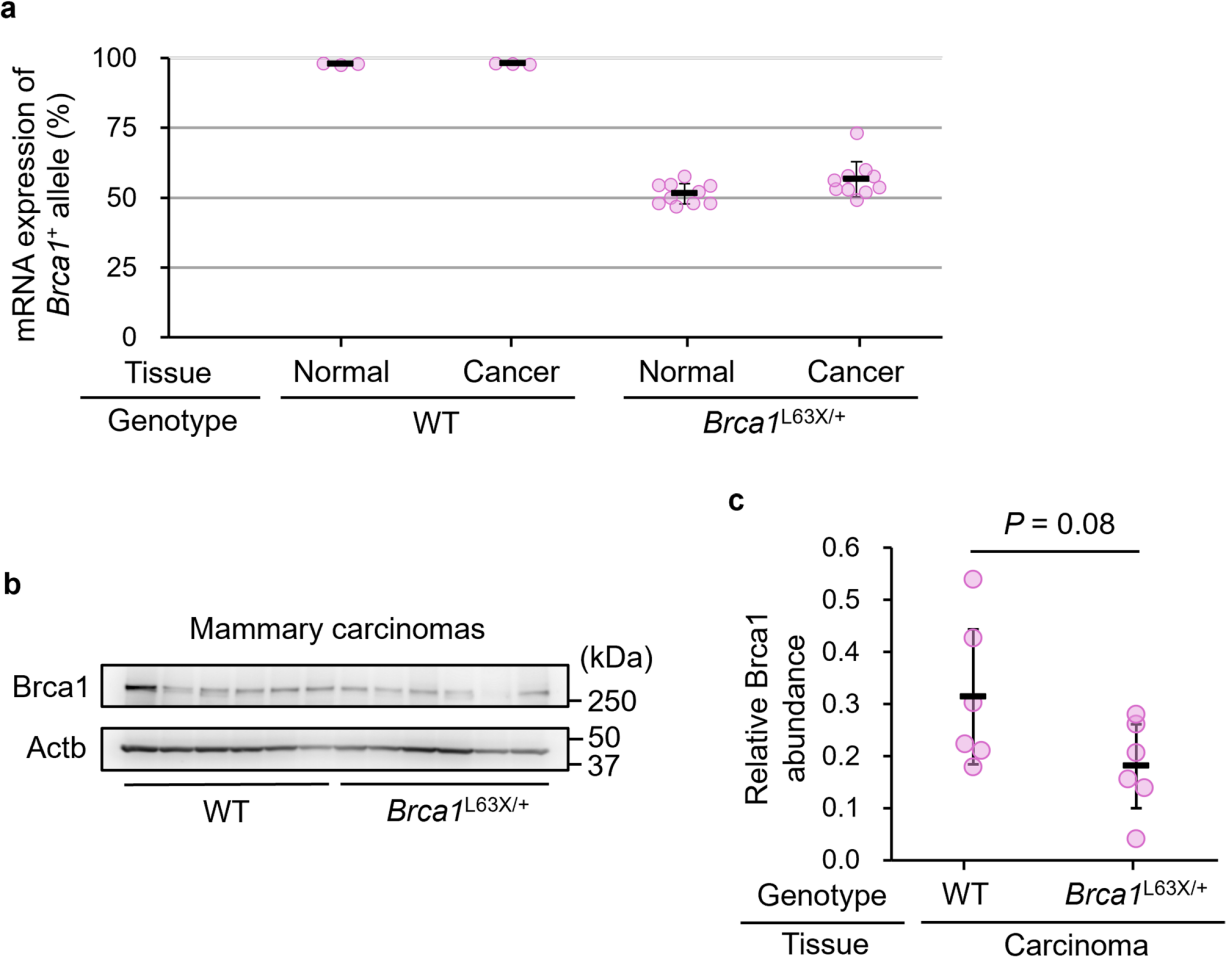
Expression of the *Brca1*^+^ allele in irradiated rat carcinomas. (**a**) The occupancy of the *Brca1*^+^ allele revealed by allele-specific RT-qPCR of normal mammary tissue and mammary carcinomas in irradiated WT and *Brca1*^L63X/+^ rats. Dots, individual data points; black horizontal line, mean; error bars, SD. (**b**, **c**) Immunoblot analysis of Brca1. Brca1 was present in mammary carcinomas that developed in irradiated *Brca1*^L63X/+^ rats. The images (**b**) were cropped, and full-length gels are included in Supplementary Fig. S2. Levels of Brca1 (**c**) are relative to that of Actb. The *P*‑value was calculated using Student’s *t*‑test.

We previously reported that the *Brca1*^L63X/+^ rat serves as an animal model of Brca1 haploinsufficiency, based on its gene dosage and increased mammary carcinoma risk without additional mutation of the wild-type *Brca1* allele^23^. In this study, to confirm retention of *Brca1* heterozygosity in tumors at the mRNA level, we quantified the ratio of *Brca1*^L63X^ to *Brca1*^+^ alleles by allele-specific RT-qPCR (Fig. 1a). The occupancy of the *Brca1*^+^ allele was 47–58% in mammary tissues and 49–73% in mammary carcinomas of *Brca1*^L63X/+^ rats, indicating that *Brca1*^+^ was maintained in tumors. In addition, Brca1 protein was expressed in tumors of irradiated *Brca1*^L63X/+^ rats, and the expression level tended to be lower in tumors from irradiated *Brca1*^L63X/+^ rats than in those from irradiated WT rats (Fig. 1b, c). These results supported that Brca1 haploinsufficiency is involved in the increased incidence of mammary carcinomas in *Brca1*^L63X/+^ rats.

### Somatic mutations of mammary carcinomas in WT and *Brca1*^L63X/+^ rats

**Fig. 2:**
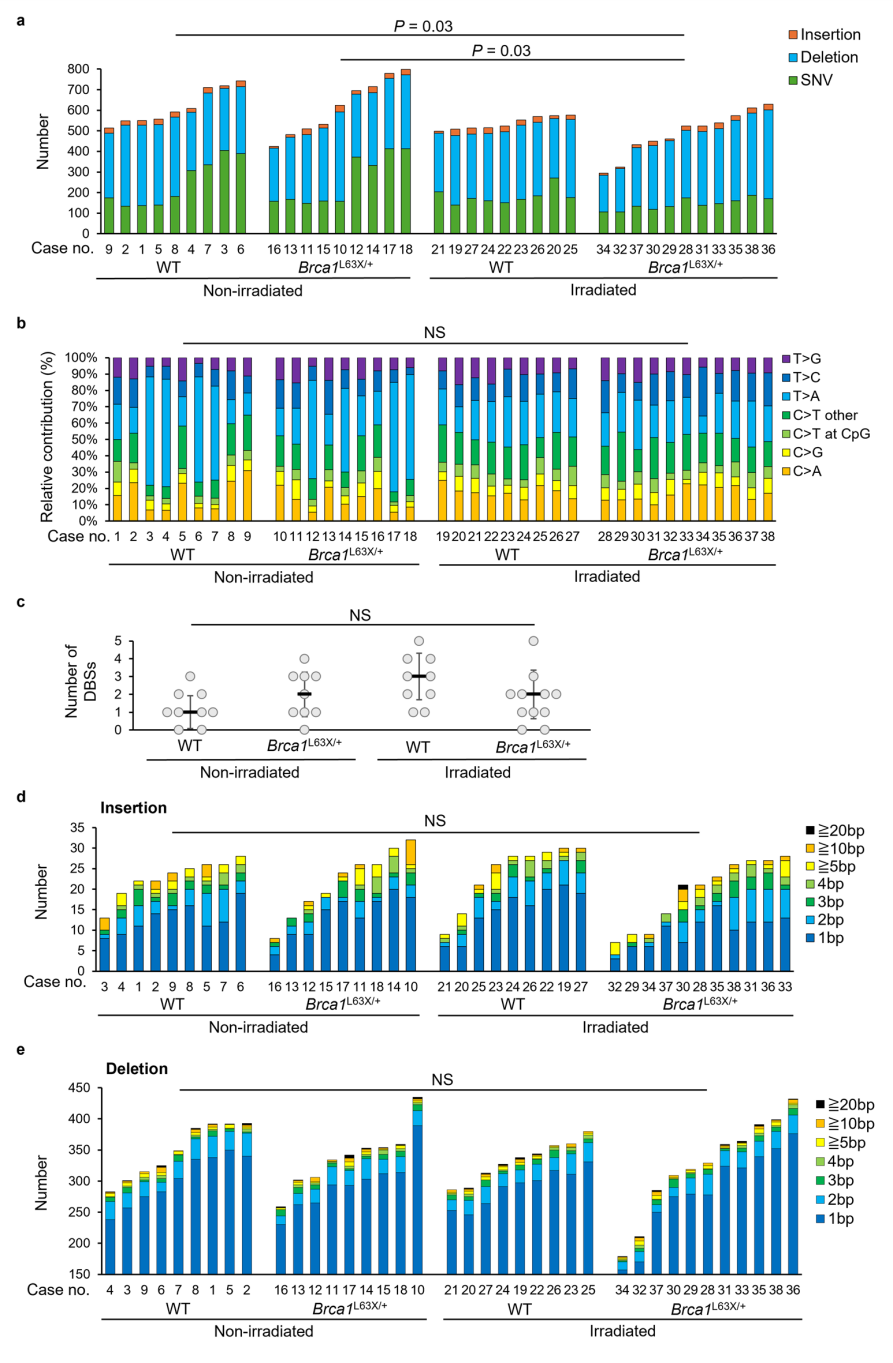
Number of somatic mutations in mammary carcinomas identified by whole-exome sequencing. (**a**) The number of SNVs, insertions, and deletions per sample. *P*-values were determined by Tukey-Kramer test. (**b**) The relative contribution of each type of SBS. NS, not significant (Tukey-Kramer test). (**c**) Number of DBSs. Dots, individual data points; black horizontal line, mean; error bars, SD. NS, not significant (Wilcoxon rank-sum test with Benjamini-Hochberg adjustment). (**d**) The number of insertions by length. NS, not significant (Tukey-Kramer test). (**e**) The number of deletions by length. NS, not significant (Tukey-Kramer test).

We found no significant differences between WT and *Brca1*^L63X/+^ rats with regard to the number of somatic mutations (i.e., those detected as SNVs and small InDels) in spontaneous carcinomas and radiation-associated carcinomas (Fig. 2a). On the other hand, the number of somatic mutations in radiation-associated carcinomas from *Brca1*^L63X/+^ rats was significantly lower than in spontaneous carcinomas from WT rat (*P* = 0.03) and *Brca1*^L63X/+^ rats (*P* = 0.03) (Fig. 2a). Next, we classified SNVs into single-base substitutions (SBSs), doublet-base substitutions (DBSs; two consecutive SNVs), and multibase substitutions (three or more consecutive SNVs), and SBSs were further divided into seven types. There were no significant differences in the number of SBSs of each type, DBSs and multibase substitutions in carcinomas, regardless of rat genotype, in both the nonirradiated and irradiated groups (Fig. 2b, Supplementary Table S2, Fig. 2c, Supplementary Table S3). The number of InDels by length was similar in spontaneous and radiation-associated carcinomas from WT and *Brca1*^L63X/+^ rats (Fig. 2d, e). A previous study reported that the deletion/insertion (DEL: INS) ratio was higher (i.e., DELs were significantly enriched) in radiation-associated second malignancies and breast tumors with germline *BRCA1*/*BRCA2* deficiency than in primary tumors^25^. Thus, we compared the DEL: SNV ratio, DEL: INS ratio, and InDel: SNV ratio in spontaneous and radiation-associated tumors between WT and *Brca1*^L63X/+^ rats and found no significant differences (Supplementary Fig. S3). The lengths of CNV regions detected in carcinomas did not differ by rat genotype (Supplementary Table S4). Biallelic copy-number loss was detected only in carcinomas of *Brca1*^L63X/+^ rats (*n* = 1 in spontaneous tumors, *n* = 1 in radiation-associated tumors; Supplementary Table S4), but these were few and showed no strong trend. In summary, spontaneous and radiation-associated carcinomas arising under Brca1 haploinsufficiency showed no marked differences compared with those of WT rats in terms of the frequency of SNVs, InDels and CNVs.

### Mutational signatures of mammary carcinomas that developed in *Brca1*^L63X/+^ rats

**Fig. 3:**
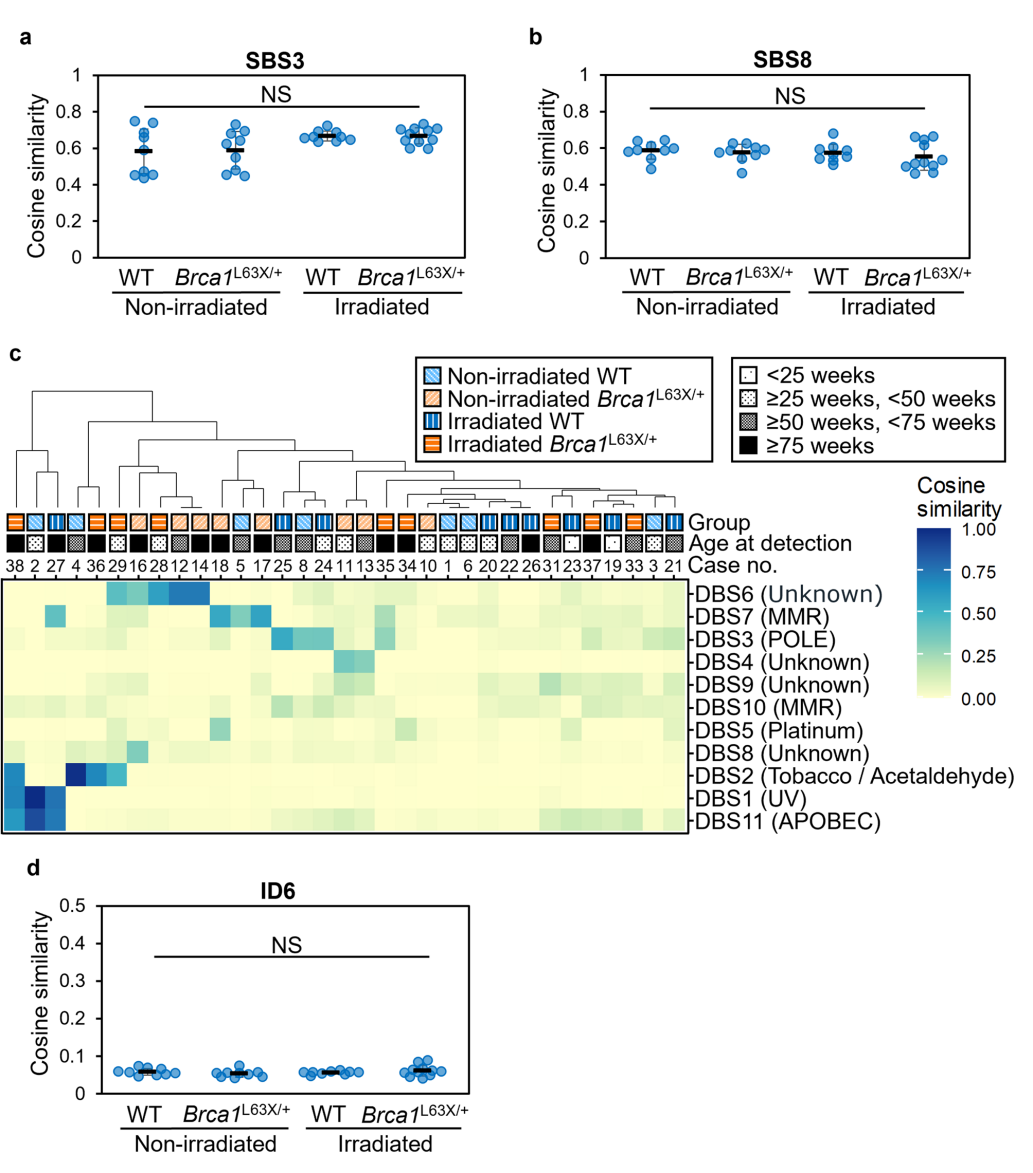
COSMIC mutational signatures in rat mammary carcinomas. Cosine similarity of (**a**) SBS3, (**b**) SBS8, (**d**) ID6. Dots, individual data points; black horizontal line, mean; error bars, SD. NS, not significant (Steel-Dwass test). (**c**) Cosine similarity of detected DBS signatures in each carcinoma.

To further characterize the mutation spectrum of mammary carcinomas, mutational signature analysis was performed on SBS, DBS and InDel profiles using the COSMIC Mutational Signatures database. There were no differences in SBS3 and SBS8, the known SBS signatures involved in *BRCA1*/*BRCA2* deficiency, between tumors from WT and *Brca1*^L63X/+^ rats (Fig. 3a, b). As shown in Supplementary Fig. S4 online, clustering based on cosine similarity to SBS signatures in individual tumors showed that cluster A consisted only of spontaneous carcinomas (8/8 tumors), cluster B had a large portion of spontaneous carcinomas (6/9 tumors), and cluster C was predominated by specimens from the irradiated group (17/21 tumors). Thus, the SBS signature spectrum in carcinomas showed variations due to radiation exposure, although no effect of Brca1 haploinsufficiency was observed. In the analysis of DBS signatures, high similarity to DBS signature 6 (DBS6) was found only in spontaneous and radiation-associated tumors from *Brca1*^L63X/+^ rats (*n* = 5, Fig. 3c). The mutational process of DBS6 is unknown in the COSMIC database but has been suggested to be related to homologous recombination deficiency^26^. For InDel signatures, all carcinomas had a similar mutation spectrum regardless of treatment or genotype (Supplementary Fig. S5), and a signature ID6, which is related to homologous recombination deficiency, was not enhanced in tumors of *Brca1*^L63X/+^ rats (Fig. 3d). Taken together, this analysis revealed DBS6 as a characteristic mutational signature of both spontaneous carcinomas and radiation-associated carcinomas of *Brca1*^L63X/+^ rats.

### Radiation-associated carcinomas arising in *Brca1*^L63X/+^ rats have fewer cancer-driver mutations than those in WT rats

**Fig. 4:**
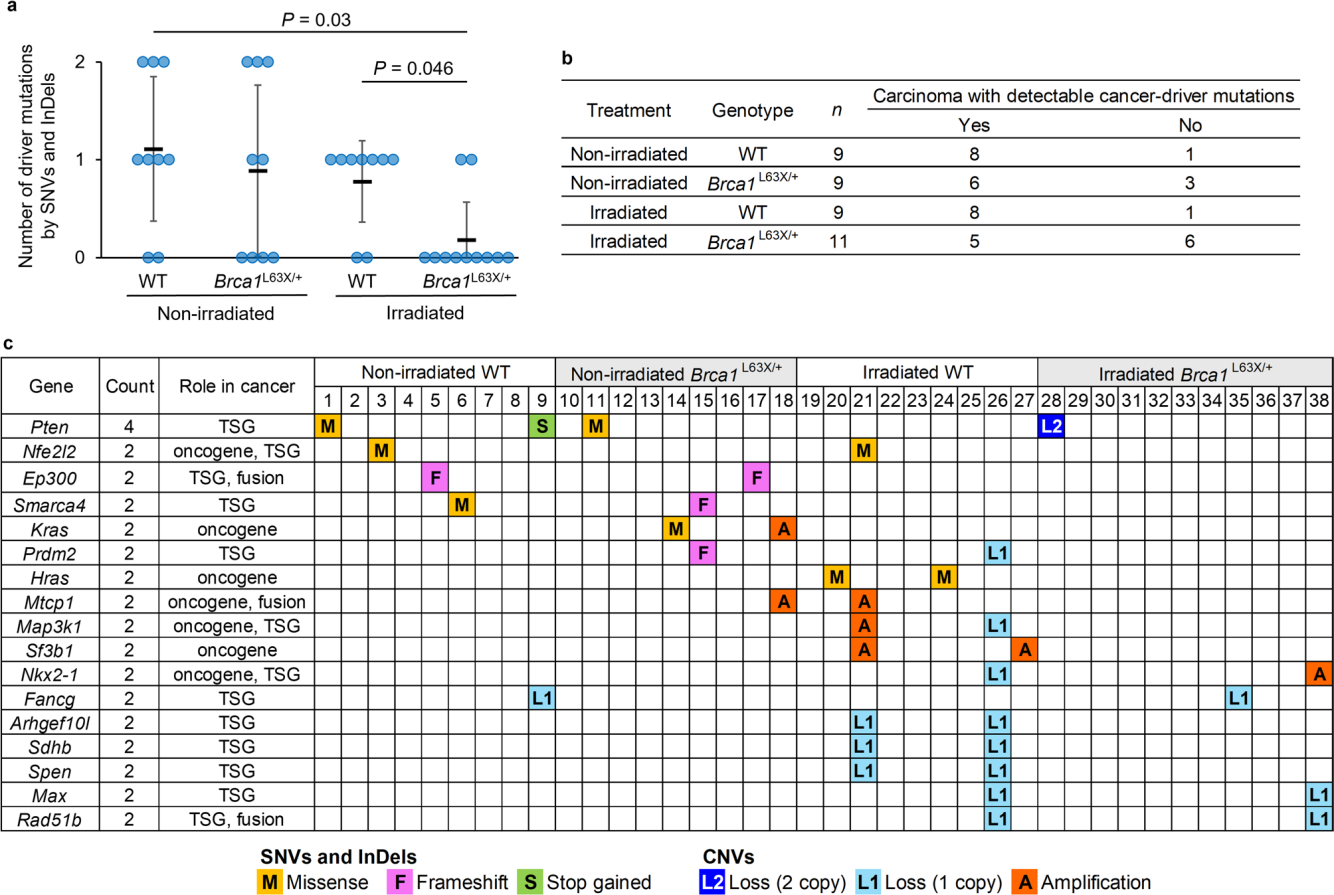
Cancer-driver mutations identified by whole-exome sequencing in rat mammary carcinomas. (**a**) Number of cancer-driver mutations induced by SNVs and InDels. *P*-values were determined by Steel-Dwass test. (**b**) Number of carcinomas having cancer-driver mutations by nonsynonymous mutations and CNVs. *P* = 0.07 (irradiated WT vs irradiated *Brca1*^L63X/+^) by Fisher’s exact test. (**c**) Cancer-driver mutations detected in multiple mammary carcinomas. Missense mutations in the protooncogenes *Kras* and *Hras* were in the codon for glycine 12, and these mutations have been established as oncogenic mutations.

To identify somatic mutations that potentially contribute to the development of mammary carcinomas, we extracted nonsynonymous SNVs/InDels—including missense, nonsense and splice-site mutations—and CNVs affecting cancer genes, and defined these mutations as cancer-driver mutations using the COSMIC Cancer Gene Census (ver. 100), a catalogue of genes with reported mutations causally implicated in cancer (Supplementary Table S5). All cancer-driver genes with nonsynonymous mutations identified in this analysis have been reported by COSMIC to be mutated in human breast cancer. As shown in Supplementary Table S5, 12 missense mutations of cancer-driver genes were detected in mammary carcinomas. In 8 of these, the same amino-acid substitutions were reported in human cancers (*Hras* missense mutation at p.G12, *Pten* at p.C136F and p.D92H, *Nfe2l2* at p.E82D and p.G31R, *Pparg* at p.D439N, *Kras* at p.G12V, and *Smarca4* at p.R885H); 2 others were found in domains important for the function of genes (*Mlh1* at p.N515H affecting the PMS2/MLH3/PMS1 interaction domain, and *Ebf1* at p.A151D affecting the DNA-binding domain), and 2 mutations (*Atm* at p.S1115I, and *Phox2b* at p.T205P) were not reported in COSMIC or did not affect a functional domain. Somatic mutations in orthologs of genes frequently reported in human breast cancer, such as *Tp53* and *Myc*, were not observed in this analysis. Because previous studies analyzing mutations in rat mammary tumors have reported that *Tp53* and *Myc* mutations are rare^27,28^, our findings likely reflect intrinsic biological characteristics of rats rather than incomplete analysis. The number of cancer-driver mutations by SNVs and InDels was significantly lower in radiation-associated carcinomas of *Brca1*^L63X/+^ rats than in those of WT rats (*P* = 0.046, Fig. 4a). Furthermore, radiation-associated carcinomas with no detectable cancer-driver mutations were marginally more frequent in *Brca1*^L63X/+^ rats than WT rats (*P* = 0.07, Fig. 4b, Supplementary Table S5). The loss of three tumor-suppressor genes (*Cdkn2a*, *Pten* and *Fas*) as a consequence of biallelic deletions was observed only in carcinomas from *Brca1*^L63X/+^ rats, but these were in 2 tumors and there was insufficient evidence to attribute them to Brca1 haploinsufficiency (Supplementary Table S5). To evaluate distinctive cancer-driver mutations in carcinomas of *Brca1*^L63X/+^ rats, we extracted mutations found in multiple tumors (Fig. 4c); no mutations were unique to carcinomas from *Brca1*^L63X/+^ rats.

## Discussion

The *Brca1*^L63X/+^ rat has been considered as a Brca1 haploinsufficiency model because they exhibited increased risk of mammary carcinomas due to reduced gene dosage without additional mutations in the wild-type *Brca1* allele^23^; however, Brca1 expression had not been examined. Here, we confirmed retention of *Brca1* expression at both the mRNA and protein levels in mammary tumors of *Brca1*^L63X/+^ rats. These lines of circumstantial evidence, together with the *Brca1* gene dosage, support the notion that Brca1 haploinsufficiency contributes to tumorigenesis in *Brca1*^L63X/+^ rats. On the other hand, although Brca1 protein levels tended to be lower in tumors from *Brca1*^L63X/+^ rats, the difference was not statistically significant. Given potential tumor‑specific gene expression changes and the inherent limitations in the quantitative accuracy of immunoblotting, it is likely that the true difference in Brca1 expression levels could not be fully captured by this analysis.

In this study, we compared WT and *Brca1*^L63X/+^ rats with regard to the mutation spectrum identified by whole-exome sequencing in spontaneous and radiation-associated mammary carcinomas and investigated the characteristic mutation spectrums inherent in *Brca1*^L63X/+^ rats. High similarity to DBS6 was observed only in *Brca1*^L63X/+^ rat carcinomas, although the number of tumors was small. A whole-genome sequencing of ovarian cancer has reported that DBS6 co-occurs with a BRCA-related mutational signature (SBS3)^26^. Therefore, our result implies that Brca1 haploinsufficiency is involved at least in part in the mutational process of DBS6. We found few carcinomas that were highly similar to DBS6, perhaps because we analyzed only exome regions; it is possible that a genome-wide mutation analysis could reveal an association between Brca1 haploinsufficiency and DBS6. Although DBS6 has been detected in breast and ovarian cancers^29^, its relevance to *BRCA1*/*2* germline mutations has not been investigated^2^; a DBS signature analysis of tumors with mono-allelic *BRCA* germline mutation is thus warranted. BRCA-associated SBS signatures^30,31^(SBS3 and SBS8) were not prominent in carcinomas of *Brca1*^L63X/+^ rats in this study. This observation is consistent with a previous report that human breast cancer with a *BRCA1*/*2* mono-allelic germline mutation did not show prominent SBS3, as seen in breast cancers with biallelic *BRCA1*/*2* inactivation^2^. These results suggest that SBS3 and SBS8 occur after LOH of *BRCA1*, and the process of BRCA1 haploinsufficiency (i.e., early phase in *BRCA*-related carcinogenesis) would lead to a different mutation spectrum.

The number of cancer-driver mutations induced by SNVs and InDels was significantly less in radiation-associated mammary carcinomas of *Brca1*^L63X/+^ rats than those of WT rats; moreover, irradiated *Brca1*^L63X/+^ rats tended to have more carcinomas with no detectable cancer-driver mutations. These results suggest that irradiated *Brca1*^L63X/+^ rats are prone to oncogenic mutations—such as structural variants (SVs) and epigenetic alterations—through mechanisms that are undetectable by whole-exome sequencing. One report described a genome-wide increase in SVs (large deletions and inversions) in second malignancies after radiotherapy^32^. In addition, excessive DNA damage and insufficient DNA repair promote the aging process, and epigenetic alterations are hallmarks of aging^33^. Consistent with this concept, one study of normal mammary epithelial cells from *Brca1* heterozygous mice showed epigenetic changes that partially mimicked those observed in mammary tumor cells, in contrast to those of WT mice^34^. Therefore, additional experiments, such as whole-genome sequencing and comprehensive epigenomic analysis, are necessary to understand whether Brca1 haploinsufficiency under radiation-induced DNA damage increases the frequency of cancer-driver mutations by SVs and/or epigenetic alterations.

A role for BRCA1 haploinsufficiency in carcinogenesis is considered to be induction of genomic instability^13^; however, our findings contradict this notion. Previous studies have indicated that non-neoplastic cells or tissue harboring a *BRCA1* heterozygous mutation exhibit increased genomic instability compared with those of WT^18,35,36^. Nevertheless, evidence has been lacking regarding genomic instability related to BRCA1 haploinsufficiency in cancer in carriers of a *BRCA1* pathogenic variant; tumors with *BRCA1*/*2* mono-allelic loss did not show a greater number of SNVs, InDels or SVs, or even an earlier age at breast cancer diagnosis, than sporadic cancers, albeit the sample size was small^2^. Consistent with these findings for humans, our results for spontaneous and radiation-associated mammary carcinomas in *Brca1*^L63X/+^ rats were equivalent to those for WT rats in terms of the frequency of somatic mutations and age at detection. Therefore, genomic instability may not contribute appreciably to the accumulation of somatic mutations in carcinogenesis with BRCA1 haploinsufficiency, although our research lacks data on SVs. Besides genomic instability, recent studies have suggested that BRCA1 haploinsufficiency promotes carcinogenesis by altering the tissue microenvironment. Cancer-associated fibroblasts were enriched in breast cancers and noncancerous breast tissue from women with germline *BRCA1* mutations compared with tissues from noncarriers^37,38^. Another study reported that mammary epithelial cells derived from carriers of mutant *BRCA1* showed rapid telomere erosion compared with WT cells, resulting in increased expression of the senescence-associated secretory phenotype factor^35^. With the above considerations, Brca1 haploinsufficiency may contribute to carcinogenesis through mechanisms other than accumulation of SNVs, InDels or CNVs (e.g. SVs undetectable by whole-exome sequencing, epigenetic alterations, or microenvironmental changes).

Taken together, this study presents novel evidence suggesting that Brca1 haploinsufficiency contributes to carcinogenesis by bypassing the generation of cancer-driver mutations that would otherwise occur via accumulation of nonsynonymous mutations and CNVs. Further elucidation of Brca1 haploinsufficiency–related carcinogenesis in terms of SVs, the tissue microenvironment, and epigenetics will be key to providing strategies of chemoprevention for carriers of a *BRCA1* germline variant.

## Materials and methods

### Tumor samples

Mammary carcinomas that developed in female WT and *Brca1*^L63X/+^ rats (Jcl:SD background; Clea Japan, Tokyo, Japan) in the previous experiment^23^ were used in this study (*n* = 38; Table S1). *Brca1*^L63X/+^ rats were generated through CRISPR-Cas9–based genome editing as described^23^. Briefly, the *Brca1*^L63X/+^ rats and WT littermates were weaned at 3 weeks of age and whole-body irradiated with 2 Gy of ^137^Cs γ rays (0.5 Gy/min) at age 3 weeks or left untreated. These rats were housed in autoclaved aluminum cages with sterilized wood chips (the number of cage companions was 2 to 3) and maintained in specific-pathogen-free rooms with controlled temperature (23 °C ± 1 °C) and humidity (50% ± 5%) under a regular 12-hour light/12-hour dark cycle with a standard laboratory diet (CE-2; Clea Japan) and chlorinated/acidified water provided ad libitum. The rats were palpated weekly, and tumors reaching ~ 2 cm in diameter were biopsied under isoflurane anesthesia (~ 2% in air) and diagnosed on hematoxylin-eosin-stained sections. Rearing, palpation, biopsy and diagnosis of biopsy specimens were done blindly for the experimental group of rats. When rats were identified as having mammary carcinoma or showed general deterioration, they were euthanized by drawing cardiac blood under isoflurane anesthesia (~ 4% in air) and autopsied; subsequently, mammary tumors were collected. We used all mammary carcinomas that were first palpated in individual rats. Cases 1–20 and 28–38 (Supplementary Table S1) were from an animal experiment described above^23^. Cases 21–27 were from another animal experiment^24^; these tumors occurred under the same irradiation conditions (2 Gy of γ-rays, 3 weeks of age) and rearing conditions as in the experiment mentioned above. Excised tumors were fixed in 10% phosphate-buffered formalin and embedded in paraffin and used for immunohistochemistry. Fresh mammary tumors and tissues stored at −80 °C were used for allele-specific reverse transcription quantitative PCR (RT-qPCR), immunoblotting, and whole-exome sequencing. Tumors from rats that died prior to euthanasia and autopsy were excluded from the analysis. All animal experiments herein were approved by the Institutional Animal Care and Use Committee (approval numbers 07-1016, 14-1021 and 16-1034) and were performed in accordance with the Regulation Concerning the Conduct of Animal Experiments, of the National Institutes for Quantum Science and Technology. This study is reported in accordance with the ARRIVE guidelines.

### Immunohistochemistry and analysis

Formalin-fixed, paraffin-embedded sections were immunostained with antibodies specific for estrogen receptor (ERα) (1:400; clone 6F11; Leica Biosystems, Nussloch, Germany), progesterone receptor (PgR) (1:400; clone SP42; Acris Antibodies, Herford, Germany), Erb-B2 receptor tyrosine kinase 2 (Erbb2) (1:150; clone e2-4001 + 3B5; Thermo Fisher Scientific, Waltham, MA, USA), and Ki-67 (1:200; clone SP6; Spring Bioscience, Pleasanton, CA, USA). Staining for Erbb2 was performed with a SuperSensitive Polymer-HRP IHC Detection System/DAB (BioGenex, Fremont, CA, USA). Stained sections were photographed with a NanoZoomer-XR scanner (Hamamatsu Photonics, Hamamatsu, Japan). Images of at least six areas (magnification, 40×) were selected from each carcinoma section, and the percentage of antigen-positive epithelial tumor cells and the Erbb2 score were determined using Patholoscope software (Mitani Corporation, Fukui, Japan). The staining intensity for membrane Erbb2 was graded from 0 to 3+ (referring to ASCO/CAP guidelines)^39^: 0, no staining; 1+, faint, incomplete circumferential membrane staining and within > 10% of tumor cells; 2+, weak-to-moderate incomplete circumferential membrane staining and within > 10% of tumor cells or strong complete circumferential membrane staining and within ≤ 10% of tumor cells; and 3+, strong complete circumferential membrane staining and within > 10% of cells.

### Allele-specific RT-qPCR

Total RNA was extracted using AllPrep DNA/RNA Micro kits (Qiagen, Venlo, Netherlands), and the quality of each prep was evaluated using a NanoDrop One (Thermo Fisher Scientific). Each cDNA was synthesized using SuperScript III Reverse Transcriptase (Thermo Fisher Scientific), random primers (Takara Bio, Kusatsu, Japan), and dNTP Mix (Thermo Fisher Scientific). Allele-specific qPCR (95 °C for 20 s, followed by 45 cycles of 95 °C for 5 s, 56 °C for 10 s, and 72 °C for 15 s) was performed using CycleavePCR Reaction Mix (Takara Bio) and the appropriate primers and probes (F1, R1, P1 and P2 in Table 2) with a LightCycler 96 thermal cycler (Roche Diagnostics, Mannheim, Germany). The ratio of *Brca1*^L63X^ to *Brca1*^+^ transcripts was calculated with reference to a calibration curve using mixtures of *Brca1*^+^ and *Brca1*^L63X^ synthetic DNA (S1 and S2 in Table 2; Eurofins Genomics, Ebersberg, Germany).

**Table 2:** Oligonucleotides for allele-specific RT-qPCR.

Target gene	ID	Type	Sequence (5′→3′)
*Brca1*	F1	Forward primer	CACACAGTGCGACCACATA
	R1	Reverse primer	TTTCAGCAGCTCTTCAACAA
	P1	Probe for *Brca1*^+^, FAM	CAC[a]AAGGAC
	P2	Probe for *Brca1*^L63X^, HEX	C[T]aAGGACACT
	S1	Standard of *Brca1*^+^	CCACACAGTGCGACCACATATTTTGCAAATTTTGTATGCTGAAACTCCTTAACCAGAAGAAAGGACCTTCCCAGTGTCCTT[T]GTGTAAGAATGAGATAACCAAAAGGAGCCTACAAGGAAGTGCAAGGTTTAGTCAACTTGTTGAAGAGCTGCTGAAAAT
	S2	Standard of *Brca1*^L63X^	CCACACAGTGCGACCACATATTTTGCAAATTTTGTATGCTGAAACTCCTTAACCAGAAGAAAGGACCTTCCCAGTGTCCTT[A]GTGTAAGAATGAGATAACCAAAAGGAGCCTACAAGGAAGTGCAAGGTTTAGTCAACTTGTTGAAGAGCTGCTGAAAAT

Uppercase letters denote deoxyribonucleotides; lowercase letters denote ribonucleotides. Nucleotides in brackets denote the location of an introduced T-to-A mutation in *Brca1*. FAM, 6-carboxyfluorescein-labeled; HEX, hexachlorofluorescein-labeled.

### Western blot analysis

Protein was extracted from bulk mammary carcinomas. Sodium dodecyl sulfate-polyacrylamide gel electrophoresis was performed with 15 µg of protein lysate from each tumor and standard of protein molecular weight (1610374, Bio-Rad, Hercules, CA, USA). After proteins were blotted onto a polyvinylidene difluoride membrane, a membrane was blocked with 2.0% fetal bovine serum in Tris-buffered saline containing Tween 20. A membrane was incubated with a polyclonal primary antibody specific for Brca1 (1:500; ab238983, Abcam, Cambridge, UK) and Actb as the protein-loading control (1:1000; clone AC-74, Merck, Darmstadt, Germany) and then reacted with a corresponding peroxidase-linked secondary antibody (anti-rabbit or anti-mouse IgG, Cytiva, Tokyo, Japan). Blots were visualized with ECL™ Prime Western Blotting Detection Reagent (Cytiva). Images were obtained with an Amersham™ Imager 600 (GE Healthcare, Chicago, IL, USA), and quantitative analysis of protein expression was performed using ImageJ (National Institutes of Health, Bethesda, MD, USA).

### Whole-exome sequencing and data analysis

Laser microdissection was performed to isolate epithelial cells from mammary carcinomas of rats using the MMI CellCut system (Molecular Machines & Industries, Eching, Germany). DNA was extracted from the carcinoma cells or corresponding normal mammary tissues using Qiagen AllPrep DNA/RNA Micro kits. DNA was quantified using a Qubit 2.0 Fluorometer (Thermo Fisher Scientific), and the quality of each prep was evaluated using a NanoDrop One. Sequence libraries were prepared using a SureSelect XT HS Enzymatic Fragmentation kit, a SureSelect XT Community Design Rat All Exon probe (designed based on the rat reference genome sequence rn6) and SureSelect XT HS Reagents (Agilent Technologies, Santa Clara, CA, USA). Exon-captured libraries were sequenced as paired-end 75-bp reads using the NextSeq 500/550 High Output kit v2 and NextSeq 550 System (Illumina, San Diego, CA, USA).

Data were processed using the pipeline prepared by Amelieff (Tokyo, Japan). The pipeline performed the following steps. The reads were trimmed by removing low-quality bases and removed if they were shorter than 32 bases or if > 80% of any individual read had a quality rating of < 20 using the QCleaner tool (ver. 4.1.0, Amelieff). The resulting reads were aligned to the rat reference genome rn6 using the Burrows-Wheeler Alignment tool (ver. 0.7.16)^40^. Duplicate reads were removed with SAMtools (ver. 1.6)^41^, and base quality recalibration and realignment around insertions/deletions (InDels) were performed using the Genome Analysis Tool kit (ver. 4.0.6.0)^42^. Somatic single-nucleotide variants (SNVs) or InDels in carcinomas were called with VarScan 2 software (ver. 2.4.4)^43^. A false-positive filter was then applied to remove sequencing- or alignment-related artifacts. Variants were annotated and the effect on coding sequences predicted using SnpEff software (ver. 4.3)^44^. Control-FREEC software (ver. 10.8)^45^ was used to identify copy-number variants (CNVs) in tumors as compared with normal mammary tissue from the same animal. Segments that exhibited CNV that was statistically significant relative to normal ploidy were extracted (*P* < 0.05, Wilcoxon and Kolmogorov-Smirnov tests). Analysis of the mutational signature in tumors was carried out using the R package MutationalPatterns (ver. 3.6.0)^46^ for somatic mutations with the variant allele frequency in ≥ 1% of tumor reads and no corresponding normal-tissue reads. Cancer-driver mutations were identified using the database of the Cancer Gene Census (ver. 100) released by the Catalogue of Somatic Mutations in Cancer (COSMIC)^47^, which required that the variant allele be present in ≥ 10% of tumor reads and that there were no representations of corresponding normal-tissue reads.

### Statistical analysis

All tests were carried out with the open-source software R^48^. Log-rank tests and Cox regression were carried out by using the “survival” package in R^49^. Steel-Dwass tests were carried out using the NSM3 package in R^50^. The Wilcoxon rank-sum test was performed using the “coin” package in R^51^. All tests were two-tailed. For all tests, a *P*-value of < 0.05 was considered to reflect a statistically significant difference between values.

## Supplementary Information

Below is the link to the electronic supplementary material.

Supplementary Material 1

Supplementary Material 2

## Data Availability

The DNA sequences generated and analyzed during the current study are available in the DNA Data Bank of Japan (DDBJ) under accession number PRJDB35787. Other data used in this study are available from the corresponding author upon reasonable request.
