# Supplementary material for: Mutational profiles of spontaneous and radiation-related mammary carcinomas in a rat model of Brca1 haploinsufficiency
Source: Sci Rep. 2026 Feb 24;16:10291. doi: 10.1038/s41598-026-41240-9 (PMC13031488; doi:10.1038/s41598-026-41240-9)
Supplement: Supplementary file 2 — Supplementary Material 2 [file 41598_2026_41240_MOESM2_ESM.pdf]

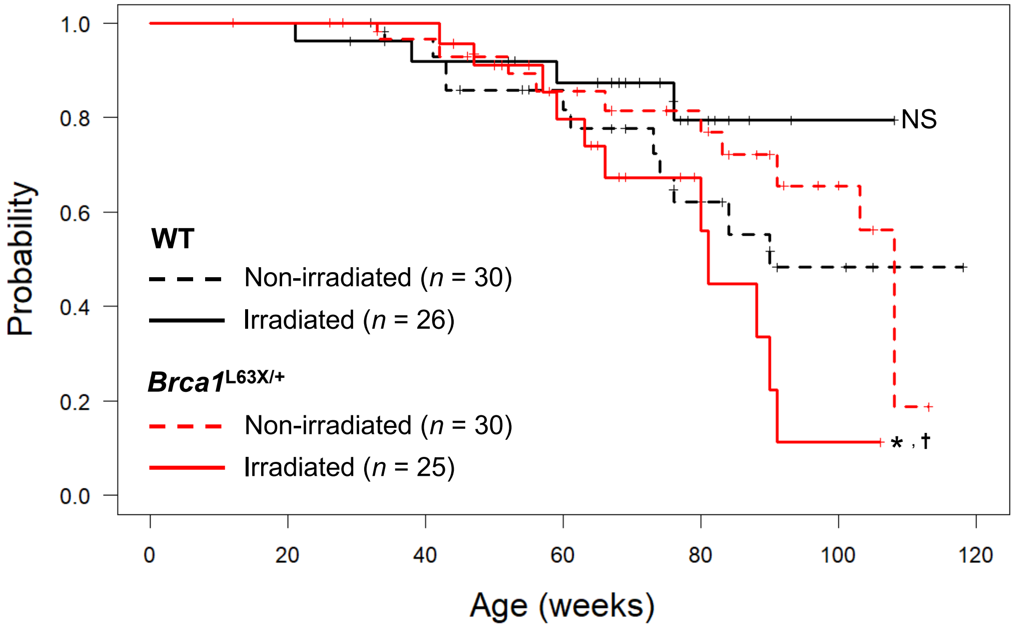

Supplementary Figure S1. Kaplan–Meier plots depicting the incidence of palpable mammary carcinoma in WT rats and *Brca1* $^{\mathrm{L63X/+}}$ rats nonirradiated or irradiated with 2 Gy at 3 weeks of age. **P* = 0.04 (compared with irradiated WT rats), log–rank test; HR = 3.1 (1.0–9.7), Cox regression. †*P* = 0.023 (compared with non-irradiated *Brca1* $^{\mathrm{L63X/+}}$ rats), log–rank test; HR = 1.7 (1.1–2.7), Cox regression. NS, not significant (*P* = 0.2, nonirradiated WT rats vs. irradiated WT rats; *P* = 0.6, nonirradiated WT rats vs. nonirradiated *Brca1* $^{\mathrm{L63X/+}}$ rats; log–rank test). *n*, total number of rats in in each experimental group. Irradiated WT rats exhibited a lower incidence of mammary tumors compared with non-irradiated WT rats. This does not reflect a protective effect of irradiation but is attributable to ovarian dysfunction induced by whole-body irradiation at 3 weeks of age[a]. Under this irradiation conditions, however, *Brca1*$^{\mathrm{L63X/+}}$ rats did not show suppression of tumor development and exhibited a higher tumor risk than irradiated WT rats (Ref 23). Thus, the reduced tumor risk by irradiation in WT rats does not influence the interpretation of the genomic analyses presented in this study.

[a] Imaoka, T. *et al*. Pre- and Postpubertal Irradiation Induces Mammary Cancers With Distinct Expression of Hormone Receptors, ErbB Ligands, and Developmental Genes in Rats. *Mol Carcinog* **50**, 539–552, doi: 10.1002/mc.20746 (2011).

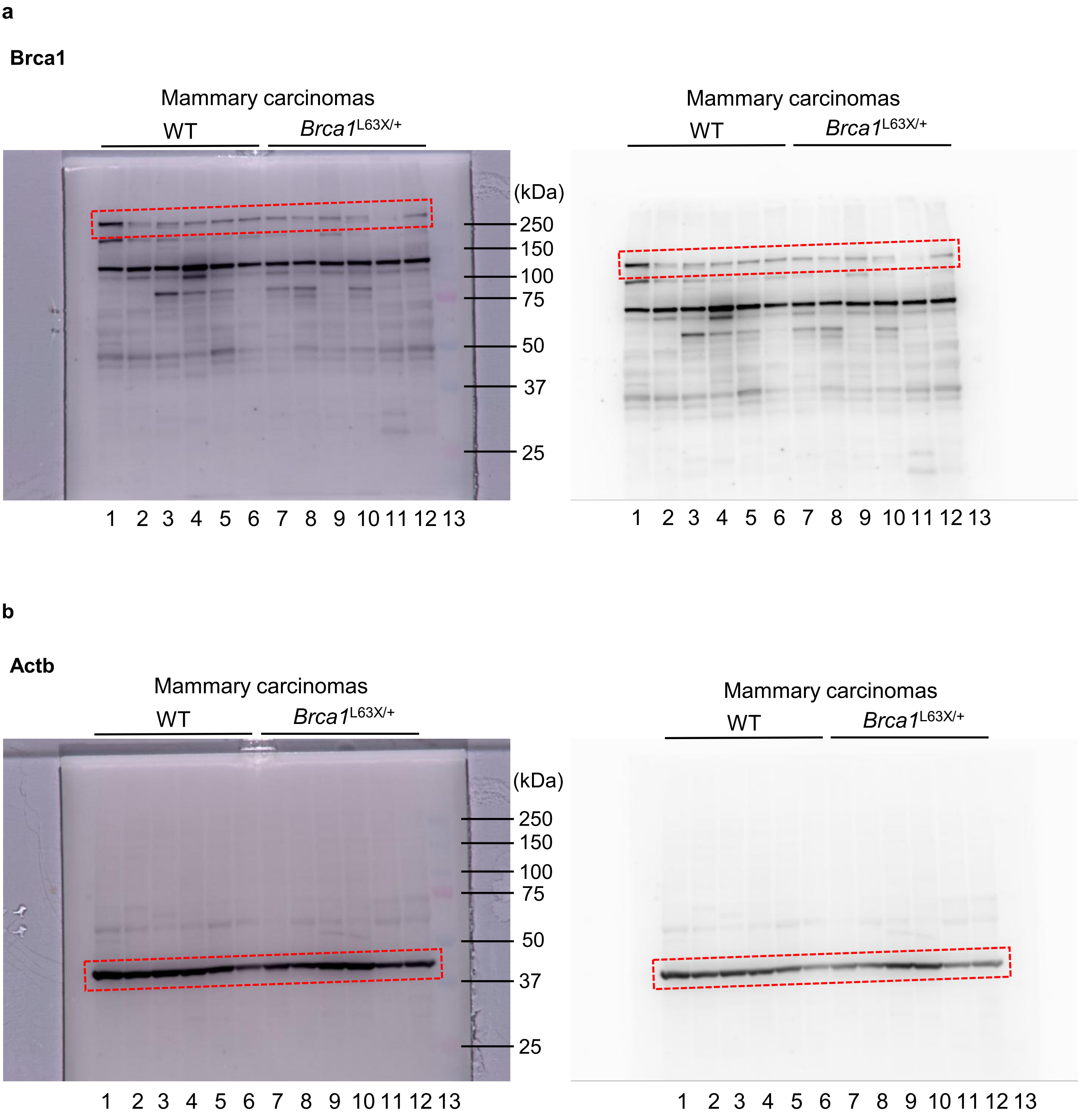

Supplementary Figure S2. Full-length western blotting images of Fig. 1b. (**a**) Brca1 and (**b**) Actb. Left panels, visualization of proteins along with molecular weight markers. Dashed lines correspond to those in the right panels. Right panels, images used for quantitative analysis. The regions delineated by the dashed lines were cropped and used in Fig. 1b. Lanes 1 to 6, mammary carcinoma from irradiated WT rats. Lanes 7 to 12, mammary carcinomas from irradiated *Brca1*$^{L63X/+}$ rats. Lane 13, molecular weight marker.

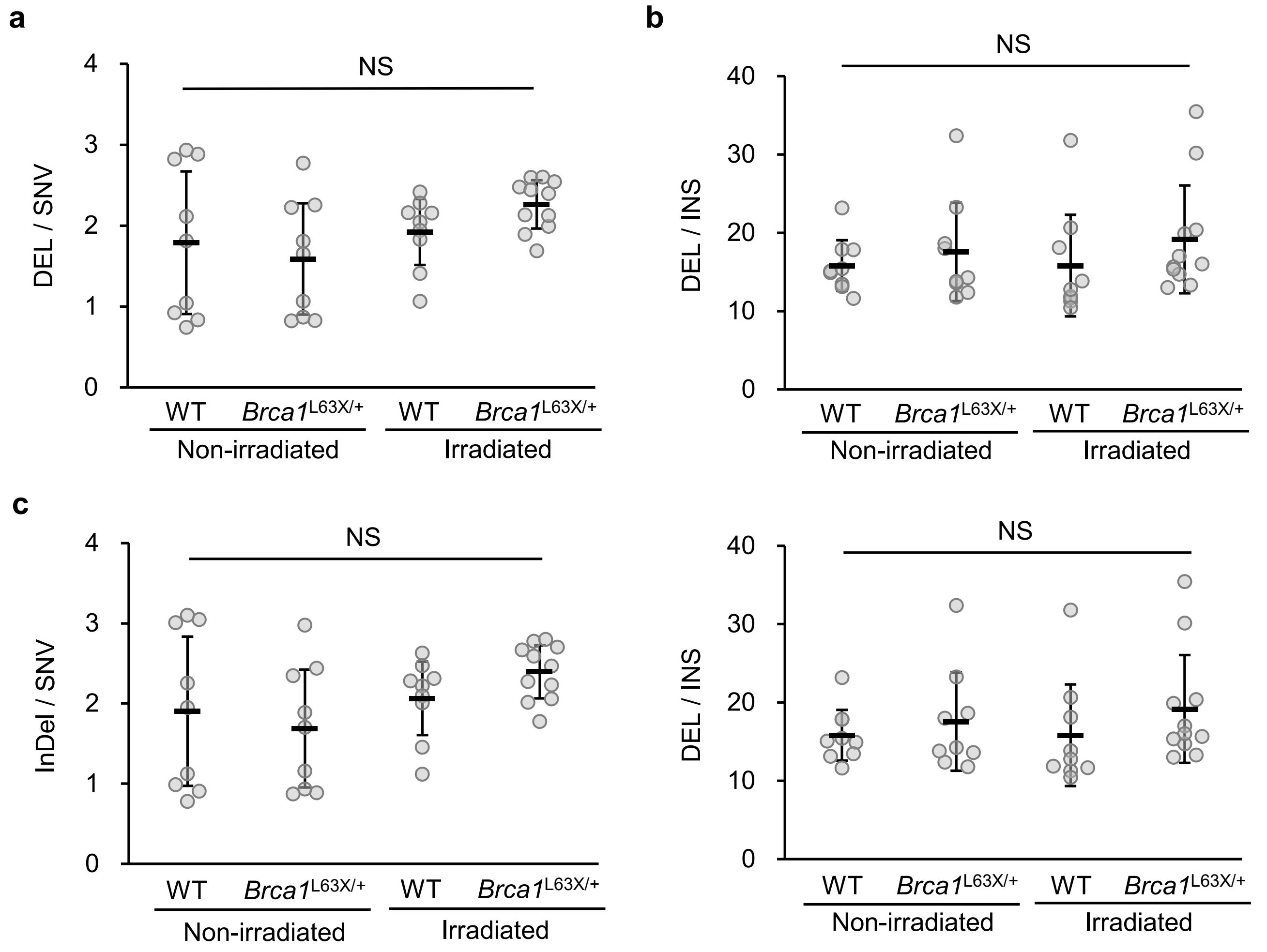

Supplementary Figure S3. Supplementary data on the number of somatic mutations. (**a**) Deletion/SNV ratio. (**b**) Deletion/insertion ratio. (**c**) InDel/SNV ratio. Dots, individual data points; black horizontal line, mean; error bars, SD. NS, not significant (Tukey-Kramer test).

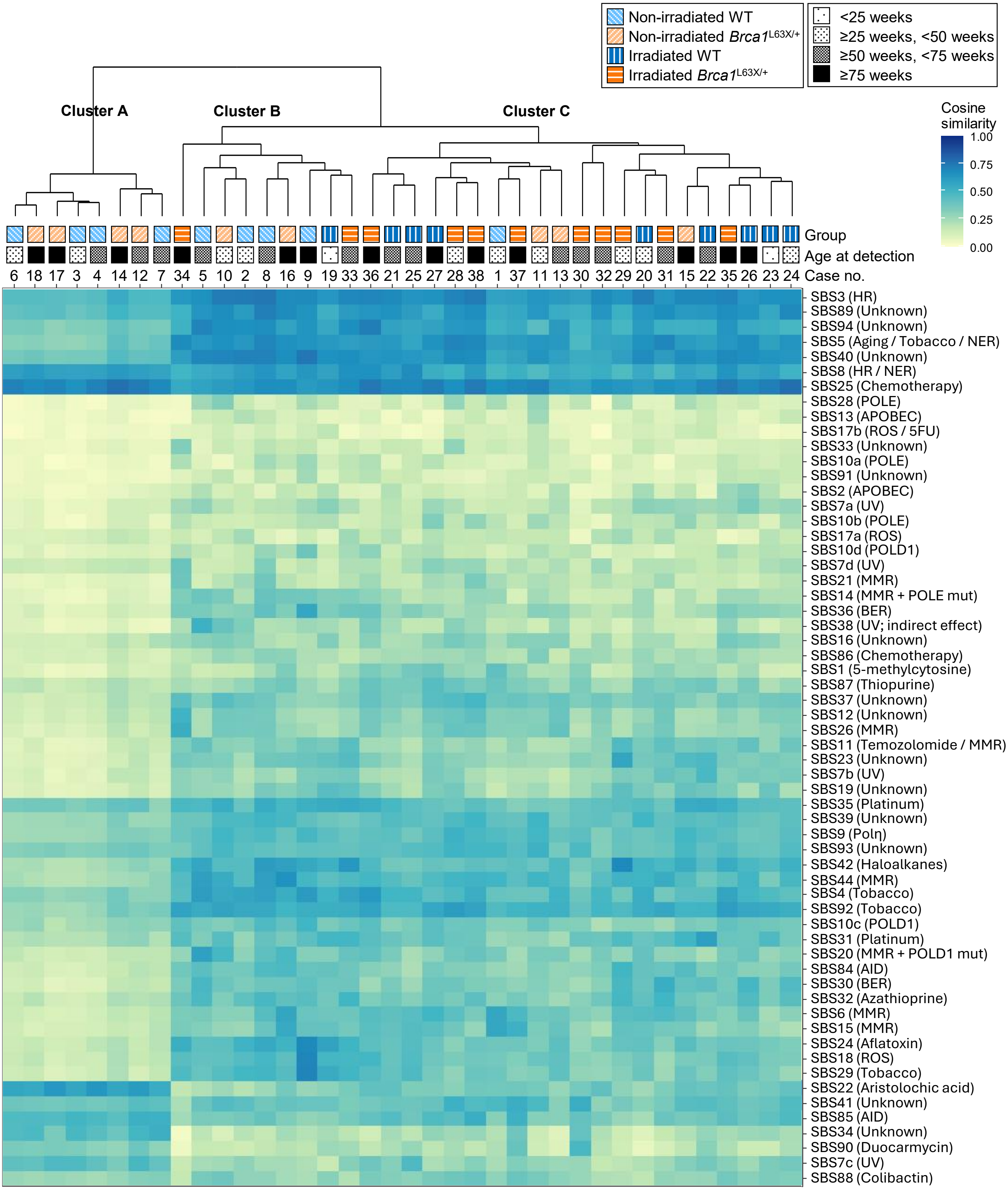

Supplementary Figure S4. Cluster classification by cosine similarity to COSMIC SBS signatures.

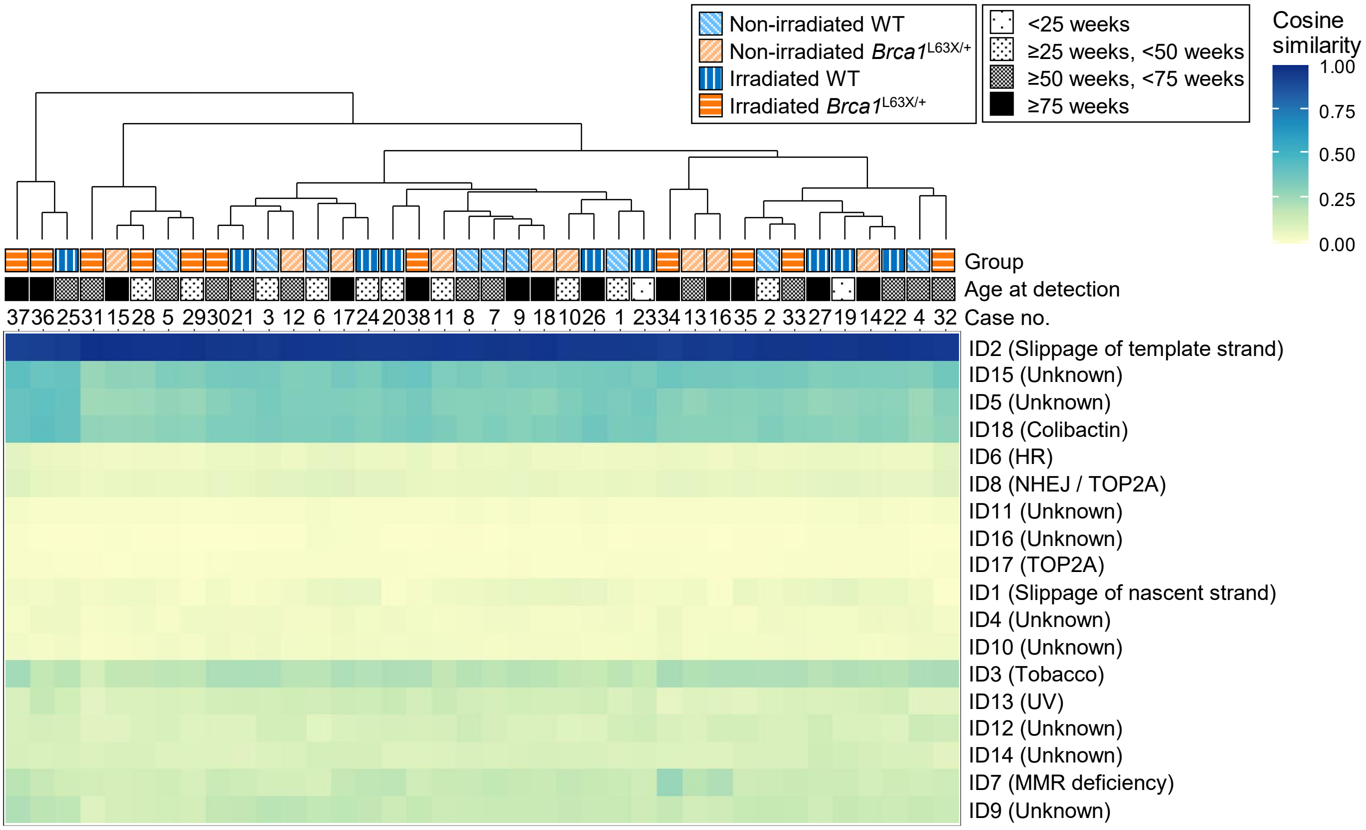

Supplementary Figure S5. Cluster classification by cosine similarity to COSMIC InDel signatures.
